# Development of Time-Resolved Fluorescence Immunochromatographic Assays for Simultaneously Detecting Tylosin and Tilmicosin in Milk in Group-Screening Manner

**DOI:** 10.3390/foods10081838

**Published:** 2021-08-09

**Authors:** Yu Wang, Jin-Yi Yang, Ying He, Lu Li, Jian-Xin Huang, Yuan-Xin Tian, Hong Wang, Zhen-Lin Xu, Yu-Dong Shen

**Affiliations:** 1 Guangdong Provincial Key Laboratory of Food Quality and Safety, College of Food Science, South China Agricultural University, Guangzhou 510642, China; xxwangyu@163.com (Y.W.); yjy361@163.com (J.-Y.Y.); heyin4102021@163.com (Y.H.); lilu5082021@163.com (L.L.); bi@stu.scau.edu.cn (J.-X.H.); gzwhongd@163.com (H.W.); jallent@163.com (Z.-L.X.); 2 Guangzhou Institute for Food Inspection, Guangzhou 510410, China; 3 Guangdong Provincial Key Laboratory of New Drug Screening, School of Pharmaceutical Sciences, Southern Medical University, Guangzhou 510515, China; tyx523@163.com

**Keywords:** time-resolved fluorescence immunochromatographic assay, tylosin, tilmicosin, milk

## Abstract

Tylosin and tilmicosin (T&T) residues in livestock products have received extensive attention from consumers. Time-resolved fluorescence immunochromatographic assay (TRFICA), as a fast, efficient and sensitive immunoassay method, has played an increasingly important role in the food safety field. Therefore, herein a quantitative and visual TRFICA was established for simultaneously detecting T&T in milk in a group-screening manner. Under the optimal conditions, the standard curve range of developed TRFICA based on the T&T was 1.87~7.47 ng/mL, and the half-maximal inhibition concentrations (IC_50_) were 4.06 ng/mL and 3.74 ng/mL, respectively. The limits of detection (LOD) of the TRFICA method were from 1.72 ng/mL to 1.39 ng/mL, and the visual cut-off values were 31.25 ng/mL and 62.50 ng/mL for T&T in milk, respectively. Moreover, the stability experiments showed that the strips could be stored at 4 °C for more than 6 months, the total detection time was less than 13 min, and the cross-reactivities (CRs) with related compounds were less than 0.1%, which concluded that the developed TRFICA method could be used in real milk sample detection.

## 1. Introduction

**Figure 1:**
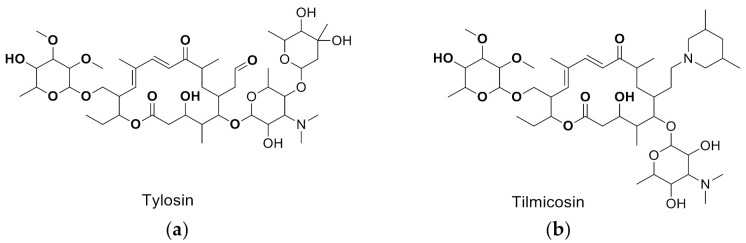
The structures of the molecules: (**a**) tylosin; (**b**) tilmicosin.

T&T are the most common premix macrolide antibiotics (Figure 1), which belong to an important class of antibiotics that inhibit Gram-positive and Gram-negative bacteria [1]. They are widely applied to animal production to prevent respiratory and enteric infections in cattle [2,3]. In addition, T&T are used as feed additives to promote animal growth and increase feed utilization [4,5], which could be accumulated as residues in the animal’s body fluids and tissues [6]. However, both of them have been banned from animal feed in the European Union since 1999 [2]. The maximum residue limits (MRLs) range from 0.05 to 1.5 mg/kg for food samples [7]. Hence, detection of residues in contaminated milk remains a major issue because of its potential risk to human health, such as allergies and drug resistance [8,9,10,11].

Multiple technologies have been applied to detect residues of T&T in food, such as high-performance liquid chromatography (HPLC), micellar electrokinetic capillary chromatography (MECC), high-performance liquid chromatography-tandem mass spectrometry (HPLC-MS/MS), and liquid chromatography-tandem mass high-resolution spectrometry (LC-HRMS) [12,13,14,15,16,17]. However, sophisticated and expensive equipment, professional personnel, and complex preprocessing methods are needed for the above methods [17,18], and existing research methods are somewhat limited in that they cannot detect multiclass residues [19]; importantly, they are not convenient for on-site diagnostics. A microbiological assay is time-consuming and has poor specificity for the target due to the interference of detection duration and the presence of active antibacterial substances (enzymes, antibodies, etc.) in milk [20,21]. Immunoassays are characterized by specificity, high throughput, and sensitivity, as well as low cost and easy operation. Enzyme-linked immunosorbent assay (ELISA) has been widely used for rapid detection of T&T in food samples [22,23,24]. Lateral flow immunoassay (LFIA) was developed to analyze six macrolide antibiotics in breast milk using latex particles labeled with BSA-clarithromycin antibody [25]. ELISA is used frequently for its high sensitivity, portability, and high throughput screening, but it was easily affected by matrix effects [26,27]. Meanwhile, previous studies have developed the ELISA method for detecting T&T simultaneously, which is cheap, has high sensitivity, large capacity, and easy operation [1,28]. Immunochromatographic assay (ICA) is a more portable and faster assay than ELISA. As labeled nanomaterials of the ICA, quantum dot embedded luminescent beads have been used to detect TYL residues in honey and milk specifically [29]. Lanthanide chelates have unique fluorescent properties, such as high quantum yields, narrow band emission peak, long stoke shift, and fluorescence lifetime. Time-resolved fluoroimmunoassay (TRFIA) has higher sensitivity than most of the conventional methods, while they decrease the matrix interference in the assay [26,30]. An Eu^3+^ fluorescent lanthanide-based TRFIA for detecting T&T in edible tissues is described, which offers cost and efficiency advantages, as well as simplified analysis steps [31]. Li et al. developed an LFIA based on the antibody-labeled time-resolved fluorescent microspheres (TRFM) as tracers; the cutoff values for T&T in milk were 2 ng/mL and 4 ng/mL, respectively [32].

In this study, a TRFICA with high sensitivity and low cost for T&T detection based on high-quality monoclonal antibody was developed successfully, the detailed conditions of Mab, coating antigen, and Immunoglobulin G (IgG) were optimized, and the standard curve of T&T was developed. The TRFICA has high stability, specificity on T&T, and neglect cross-reactivity on drugs with similar structures. The TRFICA could be used in milk detection without complicated sample preparation.

## 2. Materials and Methods

### 2.1. Materials and Instruments

Tylosin, tilmicosin, benfloxacin, enrofloxacin, azithromycin, acetylspiramycin, roxithromycin, clinfloxacin, pefloxacin, furazolidone, spiramycin, furandanone hydrochloride, and furantoin were purchased from Aladdin Reagent (Shanghai, China) Co., Ltd. Abamectin, erythromycin, florfenicol, and chloramphenicol were purchased from Energy Chemical Reagent (Shanghai, China) Co., Ltd. Florfenicolamide was purchased from Shanghai Macklin Biochemical Co. Ltd. Dimethylsulfoxide, 1-(3-Dimethylaminopropyl)-3-ethylcarbodiimide hydrochloride) (EDC), N-Hydroxysuccinimide (NHS), dicyclohexylcarbodiimide (DCC), bovine serum albumin (BSA), ovalbumin (OVA), Freund’s adjuvant (complete and incomplete), goat anti-mouse Immunoglobulin G (IgG), Casein, Trehalose, 2-(N-morpholine) ethanesulfonic acid monohydrate (MES), Tris, Proclin-300, Polyvinylpyrrolidone (PVP) and Tween-20 were purchased from Sigma-Aldrich (St. Louis, MO, USA). Monoclonal antibody was obtained from South China Agricultural University (Design of Novel Haptens and Development of Monoclonal Antibody-based Immunoassays for the Simultaneous Detection of Tylosin and Tilmicosin in Milk and Water Samples, Biomolecules, 2019, 9(12): 770). Time-resolved fluorescent microsphere (catlog: FT02C, diameter: 100 nm, laser: 360 nm, emission: 610 nm, surface function: COOH) was purchased from Suzhou Vdo Biotech Co., Ltd. (Suzhou, China). Nitrocellulose membrane, sample pad (8694), and adsorbent pad (H-8) were from Shanghai Jieyi Biotechnology Co.,Ltd (Shanghai, China). The ELISA kit for tylosin analysis was obtained from Beijing Kwinbon Biotechnology Co., Ltd. (Beijing, China). Female BALB/c mice were purchased from Guangdong Medical Laboratory Animal Center (Guangzhou, China). Buffers were prepared in our laboratory. Solution A: 0.025 M, pH 6.5, 2-morpholinoethanesulfonic acid buffer. Solution B: 0.5 g/L Casein, 0.05% Proclin-300, and 2 g/L BSA in 0.05 M, pH 8.0 Tris-HCl buffer. Solution C: 0.05 M, pH 6.5 borate buffer. Solution S: 20 g/L Trehalose, 0.2% Tween-20, 0.5% Proclin-300, 2 g/L BSA in 0.05 M, pH8.0 Tris-HCl buffer. Sample pad pretreatment solution: 0.05% Tween-20, 0.025 M PBS (pH 7.4), 0.5 g/L PVP, 15 g/L sucrose, 0.02% S9 surfactant. All other chemicals were purchased from Guangzhou chemical reagent Co., Ltd. (Guangzhou, China) and were of analytical grade or better, which the percentage concentrations were defined by weight unless otherwise specified. HM3030/HM3035 Dot Dispenser and Programmable strip cutter ZQ3500 were purchased from Shanghai Kinbio Tech.Co., Ltd. (Shanghai, China). U3010 Trace Ultraviolet Visible Spectrophotometry, Multiskan MK3 Microplate Reader, Nano Drop 2000C ultra-violet spectrophotometer were purchased fromThermo Scientific (Waltham, MA, USA). DEM-3 automatic plate washer (Top Analytical Instruments Co., Ltd., Beijing, China), time-resolved immunochromatography reader (Nanjing Microdetection Bio-Tech Co., Ltd.) and AB SCIEX 5500 triple quadrupole mass spectrometer (AB SCIEX, Redwood City, CA, USA) were used.

### 2.2. Activation and Coupling of TRFM

A total of 100 μL TRFM that was modified with carboxyl groups was centrifuged at 1.37 × 10^6^ g for 20 min. The fluorescent microspheres were evenly dispersed in 400 μL of solution A by ultrasonic stirring for 4 min after the supernatant was removed. A total of 50 mg/mL EDC and NHS mixed solution was prepared with solution A, 10 μL of which was added to activate the carboxyl group on the fluorescent microsphere particles for the convenience of antibody coupling. The above solution was fully mixed with ultrasonic stirring for 4 min and shaken for 30 min. The mixed solution was centrifuged at 1.37 × 10^6^ g for 20 min; then, the supernatant was removed. The above precipitation was remixed with 400 μL of solution C by ultrasonic stirring for 4 min, and the supernatant was removed by centrifugation (1.37 × 10^6^ g, 8 °C, 15 min). A total of 400 μL of solution C was added to the above precipitation by ultrasonic stirring for 4 min. An equal volume of monoclonal antibody was added to the redissolved solution and vortexed for 2.5 h. The supernatant of solution was removed by centrifugation (1.37 × 10^6^ g, 8 °C,15 min), and 400 μL of solution B was added to seal the non-specific binding sites on the surface of the fluorescent microspheres, sonicated for 4 min and shaken for 1 h, then removed the supernatant by centrifugation (1.37 × 10^6^ g, 8 °C, 15 min). Finally, 400 μL of solution S was added with ultrasonic stirring for 4 min, then was stored in the dark at 4 °C for further use.

### 2.3. Optimizing the Amount of Monoclonal Antibody Conjugated to Fluorescent Microspheres

Europium-chelated fluorescent microspheres with carboxyl activated group were used to conjugate the monoclonal antibody. Based on the existing research results [33,34], the amount of activated microspheres (10%, 100 nm) was fixed at 25 μL, and the amount of conjugated monoclonal antibody was optimized at 5–100 μg according to the quality of different antibodies. The different standard curves were established based on the fluorescent microspheres with different monoclonal antibodies. The antibody dosage was optimized and screened by comparing the color of the test strip and the IC_50_ of developed standard curve.

### 2.4. Selection of Microsphere-Labeled Antibody

An appropriate volume of fluorescent microsphere-labeled antibody was added in microwell plate to react with the target analyte. Excessive labeled antibodies would make the absorbance value too large and reduce its sensitivity. The detection standard curves were established based on different volumes of labeled antibodies, and the best conditions were comprehensively selected by comparing IC_50_, LOD, and color development.

### 2.5. Selection of Coating Antigen on Test Lines

Coating antigen was diluted with PB (0.02 M, pH 7.4) and sprayed onto the bottom of nitrocellulose membrane as the test (T line) by Dot Dispenser, and then dried in oven at 37 °C for 6 h. The detection standard curve was established with different coating antigen concentrations, and the best coating antigen condition was optimized combination with low IC_50_, moderate color, and less amount of coating antigen.

### 2.6. Selection of Goat Anti-Mouse IgG on Control Lines

Under the optimal conditions of coating antigen, goat anti-mouse IgG was diluted with 0.02 M, pH 7.4 PB and sprayed onto the top of nitrocellulose membrane as the test (C line) by Dot Dispenser, and then dried in oven at 37 °C for 6 h. The detection standard curve was established with different amounts of goat anti-mouse IgG. The amount of goat anti-mouse IgG was optimized combination with lower IC_50_ and the release of the labeled antibody on the NC membrane.

### 2.7. Principle and Procedure of the TRFICA

In this case, the TRFICA adopts the micropore method. The detection principle is to mix the antibody-conjugated microspheres and the sample buffer solution for 5 min at room temperature and then insert the test strip into the micropore. The test strip was put into the cartridge for reading after reaction 8 min. The color intensities on the test lines were tested for using TRFICA reader device. Take B/B_0_ as the y-axis (B_0_ is the T/C value when no drug is added, and B is the T/C value when the drug concentration is x), and the concentration of the substance to be tested was the x-axis, then, The detection standard curve of the TRFICA was established though sigmoidal fitting with a four-parameter logictic function. The calculation method as follows: the LOD (the drug concentration when B/B0 = 0.1), IC_50_ (the drug concentration when B/B_0_ = 0.5), the range of detection (the drug concentration when B/B_0_ = 0.2~0.8), and the visualization value under UV light (cut-off value).

### 2.8. The Specificity of Developed TRFICA

The specificity of test strips was determined in a cross-reaction study with related compounds. The standard solutions of tylosin, tilmicosin, benfloxacin, enrofloxacin, azithromycin, acetylspiramycin, roxithromycin, clinfloxacin, pefloxacin, furazolidone, spiramycin, furandanone, and furantoin were prepared at different concentrations, respectively, and were tested by the optimal developed TRFICA. The values of cross-reactivity (CR) were determined by CR (%) = (IC_50_ of desmycosin / IC_50_ of competitor) × 100%.

### 2.9. The Stability of Developed TRFICA Strip

Generally, low temperature and dry conditions are conducive to the preservation of immunoassay products; thus, the active coating antigen of TRFICA strip and the monoclonal antibodies of microspheres are both greatly affected by storage conditions. The stability of TRIFCA strips was evaluated by the thermal stability experiments. According to Zhang et al. [35], the storage of immunoassay products at 37 °C for 7 days is equivalent to that at 4 °C for 6 months. The prepared TRFICA strips and labeled microspheres were stored in a sealed bag together with the desiccant and then stored in a 37 °C dry box. The stability of the TRFICA strip could be judged by visual observation and the reading of the reader during the 1, 3, 5, and 7 days intervals, respectively.

### 2.10. Sample Pretreatment Method

The matrix effects of sample were eliminated by optimizing the best dilution, which was confirmed by comparing the standard curves of the diluted sample solution and PB. The milk sample matrix with T&T as the test object was directly diluted 2 times with 0.02 M PB.

### 2.11. Recovery of the Developed TRFICA for Spiked Samples

The accuracy and precision of developed method were represented by the recovery and the coefficient of variation (CV), respectively. According to the IC_50_, LOD, and LOQ of the standard curve of the established method, the recovery and CV were tested by spiking the tylosin or tilmicosin in milk with the high, medium, and low levels, respectively. The T/C values of spiked samples were calculated using the established TRFICA, and the following formula to calculate the recoveries: (concentration measured/concentration spiked) × 100. The coefficient of variation (CV) was calculated using the equation: (standard deviation/mean). Three replicates were performed for each concentration (*n* = 3).

### 2.12. Comparison of the TRFICA with ELISA and HPLC-MS/MS

To further confirm the capability and accuracy of the developed TRFICA, a comparison between TRFICA and commercial ELISA kit was conducted by using the spiked milk samples. The milk samples were from Guangdong Wen’s Food Group Co., Ltd. The sample preprocessing of ELISA analysis was performed according to the procedure of the ELISA kit instruction manual. Linear regression between the TRFICA and the ELISA was used to evaluate the consistency of these methods. In order to testify the verification of the developed TRFICA, the real samples were randomly selected and analyzed using the TRFICA and the HPLC-MS/MS, respectively.

## 3. Results and Discussion

### 3.1. SelectionPreparation of the MAb Labeled Fluorescent Microspheres

**Figure 2:**
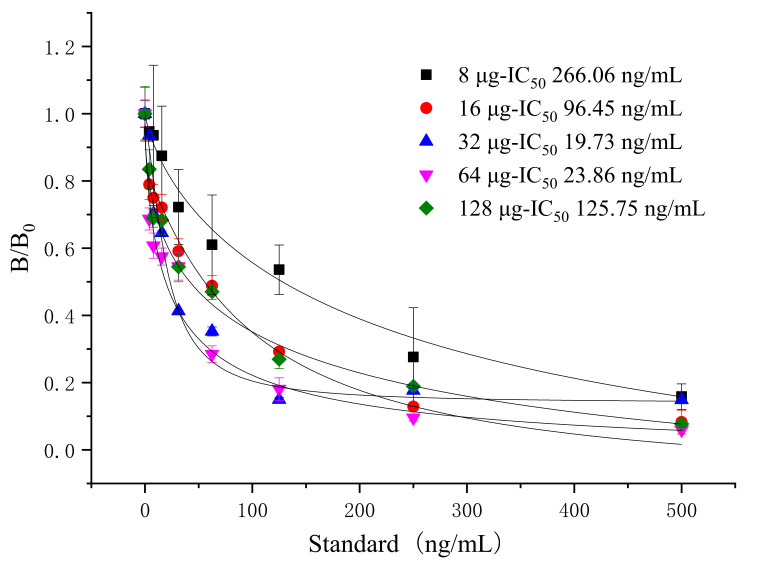
Selecting the amount of MAb for tylosin detection (*n* = 3).

**Figure 3:**
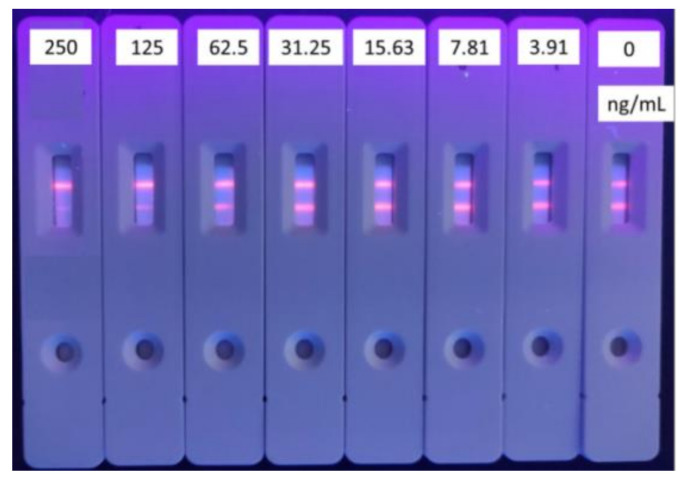
Standard curve for tylosin with the amount of MAb 32 μg (*n* = 3). (Dosage of coating antigen and goat anti-mouse IgG are 1 mg/mL and 0.093 mg/mL, respectively.)

The surface of the fluorescent microspheres is modified with carboxyl groups, which can be coupled with MAb through covalent reactions such as classic active ester reactions. The proper amount of antibodies is important for the detection because a shortfall of antibodies could cause the coagulation of the microspheres, and excess antibodies can increase the cost of the product. Thus, it is necessary to optimize the amount of MAb labeled with fluorescent microspheres. The MAb stock solution (8.0 mg/mL) was diluted to different amounts of antibodies (8, 16, 32, 48, 96 μg), then designed to couple with 2 μL of the microspheres. The standard curves were established with different amounts of microsphere-labeled antibodies (Figure 2). When the amount of MAb was 32 μg, the IC_50_ was the lowest, the sensitivity was higher, and the color of the test strip was appropriate (Figure 3).

### 3.2. Selection of the Amount of Microsphere-Labeled Antibody

**Figure 4:**
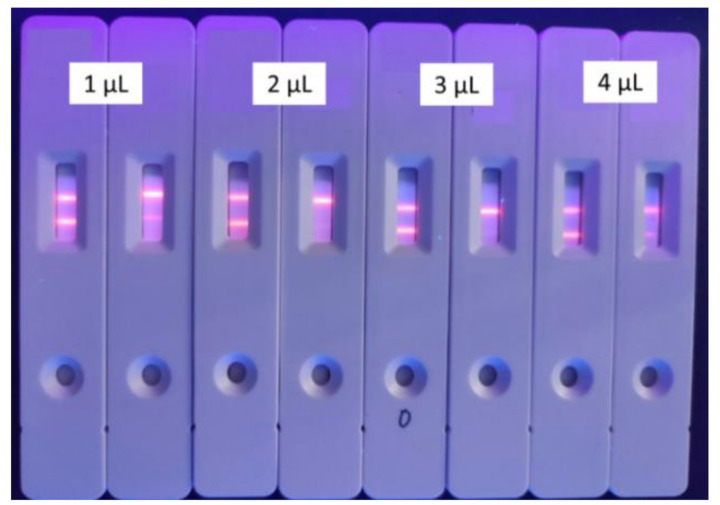
Dosage selection of the labeled antibodies for tylosin (*n* = 3). (Concentration of coating antigen and second antibody are 1 mg/mL and 0.093 mg/mL.)

Excessive microsphere-labeled antibodies will be captured by T-line and C-line, which will cause inaccurate T/C readings and poor sensitivity. Conversely, less microsphere-labeled antibodies will result in an inaccurate T/C value because of the incomplete combination between the substance and the antibody. Here, 100 μL tylosin standard solution (125 mg/mL) was reacted with 1, 2, 3, 4 μL of labeled antibody (32 μg/6μL) in a microwell plate, respectively. Although the cost and the use of antibodies were higher than that of a typical gold-nanoparticle-based strip, the proposed TRFICA strip is still valuable and has potential in practice use since it can be used as a quantitative determination. The optimal amount of microsphere-labeled antibodies were selected by calculating the respective inhibition rates. As shown in Figure 4 and Table 1, there were residual fluorescent microspheres on the NC membrane of the test strip under the amount of 1 μL and 2 μL microsphere-labeled antibody, and the dirty background can easily cause reading interference while the inhibition rate is lower. The results from the time-resolved immunochromatography reader showed that the inhibition rate was the highest when the amount of microsphere-labeled antibody was 3 μL (16 μg), while the NC membrane of the test strip was released clear.

### 3.3. Selection of the Amount of Coating Antigen on Test Line

**Figure 5:**
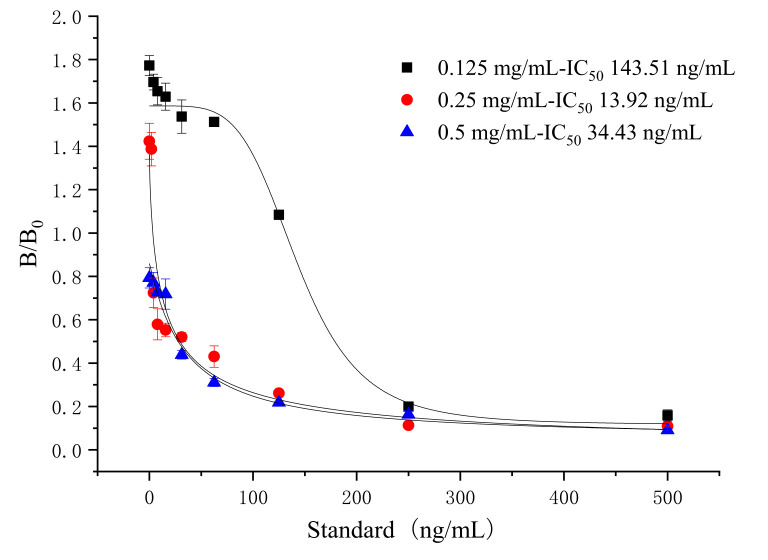
Dosage selection of the coating antigen for tylosin (*n* = 3).

**Figure 6:**
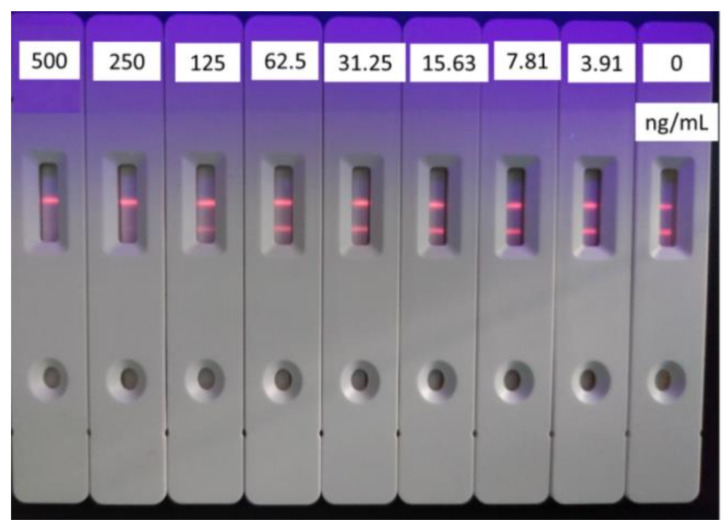
Standard curve for tylosin with coating antigen of 0.25 mg/mL (*n* = 3). (The concentration of second antibody is 0.093 mg/mL.)

The effective combination between the antigen and antibody was affected by the concentration of coating antigen of T&T on the test line (T line), which in turn affected the sensitivity. Based on the optimized dosage of antibody and labeled antibody, a standard curve was established with test line by a series concentration of coating antigen (0.125, 0.25, and 0.5 mg/mL), respectively. The IC_50_ value was the lowest when the concentration of coating antigen was 0.25 mg/mL (shown in Figure 5). Meanwhile, the fluorescent test strip was suitable for color development under ultraviolet light, and the chromatographic background was clean (Figure 6). Finally, the concentration of 0.25 mg/mL was chosen as the optimal concentration of the coating antigen on the T line.

### 3.4. Selection of the Amount of Goat Anti-Mouse IgG on Control Line

**Figure 7:**
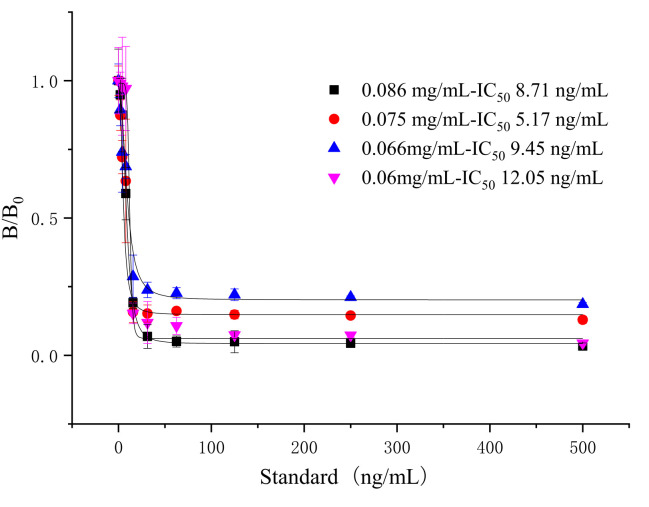
Dosage selection of the goat anti-mouse IgG for tylosin (*n* = 3).

**Figure 8:**
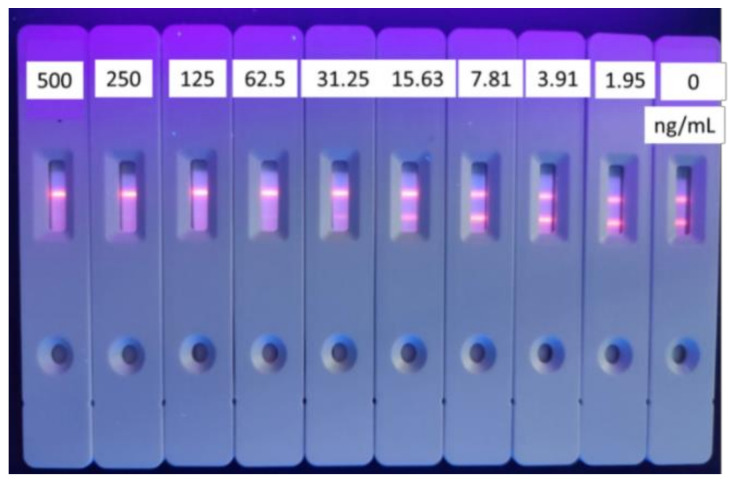
Standard curve for tylosin with goat anti-mouse IgG dosage of 0.075 mg/mL (*n* = 3).

A quantitative concentration of goat anti-mouse IgG as coating antigen on the control line (C line) was used to determine the validity of the test strip. The concentration of coated antigen on the control line would cause the color to be too deep or pale, which would lead to an inaccurate T/C value or invalid results. As shown in Figure 7 and Figure 8, the optimized concentration of goat anti-mouse IgG is 0.075 mg/mL, the IC_50_ was 5.17 ng/mL, and the fluorescent test strip was clear.

### 3.5. Cross-Reactivities of TRFIA

**Figure 9:**
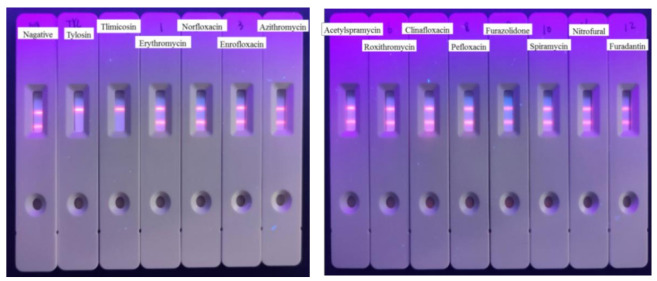
CRs of related or similar compounds in the TRFICA (*n* = 3).

Then, the specificity of the TRFIA method for the detection of tylosin was further evaluated. The compounds (as shown in Table 2) with similar chemical structures to T&T were chosen, and their crossover rates were calculated. As shown in Figure 9 and Table 2, all the selected compounds (200 ng/mL) were negative, while the T&T were positive. The TRFIA results indicated their excellent specificity on T&T (100%); they had no crossover rate with other similar compounds, which could meet the demand for the detection of T&T simultaneously.

### 3.6. Detection of T&T in Milk Using the TRFIA Method

**Figure 10:**
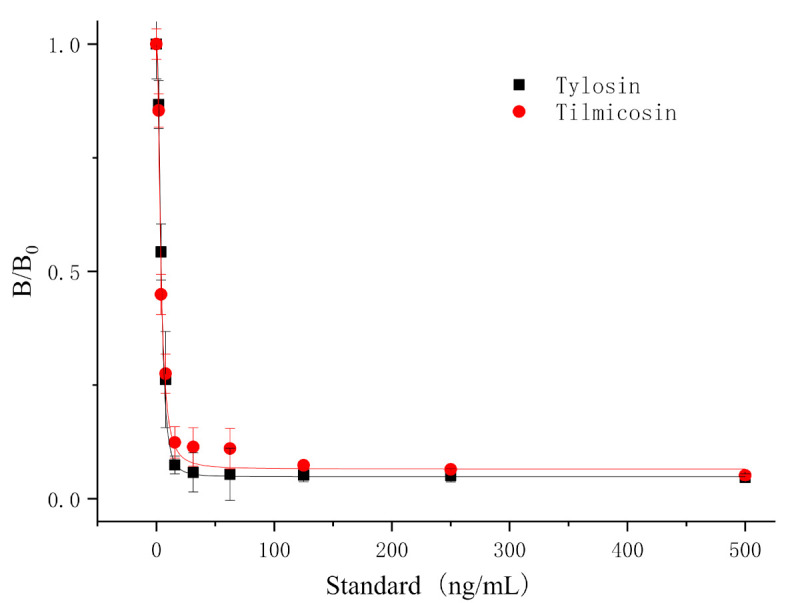
Standard curves for T&T in milk using TRFICA (*n* = 3).

The optimized TRFIA method (25 μL fluorescent microspheres (10%, 100 nm) labeled Mab, coating antigen (0.25 mg/mL) on T line, IgG (0.075 mg/mL) on C line) was applied for detecting T&T in milk. Since milk contains a large amount of protein and fat, the direct application of milk samples will cause greater matrix interference and affect the sensitivity of the method. Hence, the milk was diluted two times by 0.02 M PB and then loaded with TRFICA. The established standard curve of T&T is shown in Figure 10. The IC_50_ of this method to detect tylosin in milk matrix was 4.06 ng/mL, the detection range was 2.35~7.05 ng/mL, the LOD was 1.72 ng/mL, and the cutoff value was 31.25 ng/mL; the IC_50_ for tilmicosin was 3.74 ng/mL, the detection range was 1.87~7.47 ng/mL, the LOD was 1.39 ng/mL, and the cutoff value was 62.5 ng/mL.

In addition, we also evaluated the stability of the TRFICA test strip chromatography. The tylosin (diluted with 0.02 M PB, 200 ng/mL) was tested using TRFICA test strips stored in a drying cabinet for 1, 3, 5 days, respectively. The results are shown in Table 3. The results of the four measurements were similar and the sensitivity remained unchanged, indicating that the TRFICA test strip had a better stability rate and could be stored for more than 6 months at 4 °C.

**Figure 11:**
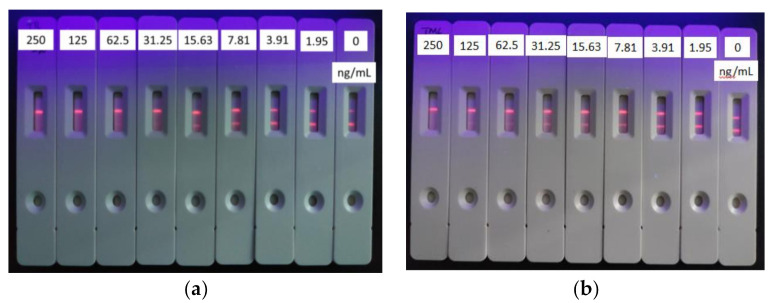
Standard curves for tylosin (**a**) and tilmicosin (**b**) in milk using TRFICA (*n* = 3).

The developed standard TRFICA method in this study had high stability, sensitivity, and specificity on the milk matrix. The TRFICA test strip chromatography results showed appropriate color depth, no obvious drag mark, and no matrix interference after diluting milk two times (Figure 11). The TRFICA develops a simple preprocessing milk method, which can meet the national milk limit requirements and is suitable for large-scale screening of T&T in milk samples.

### 3.7. Test in Spiked Samples and Real Positive Sample

To appraise the reliability of these results, all tylosin-and tilmicosin-free samples were used to verify the developed TRFICA and the commercial ELISA kit by calculating recovery rate and CV. Serial concentrations of T&T standard solutions were spiked in the negative milk samples at 5 ng/mL, 10 ng/mL, and 20 ng/mL (diluted two-fold for direct addition). As shown in Table 4, the recoveries of T&T ranged from 80.6% to 124.5% for TRFICA with CVs less than 15%. Compared with the results of TRFICA and ELISA, the developed TRFICA had a high recovery rate and good precision, which could meet the detection requirements of actual samples.

Besides, there were two positive samples with tylosin residues in three real blind samples, and they were confirmed by National Standard HPLC-MS/MS, and the determination results of real positive samples by TRFICA were also consistent with the determination results of HPLC-MS/MS (Table 5). The results indicated that the developed TRFICA could be used for the determination of T&T simultaneously in real milk samples with high accuracy.

## 4. Conclusions

TRFICA for detecting T&T in milk was established in this study. The detection condition was optimized, and the developed standard curves of TRFICA based on the T&T matrix calibration ranged from 1.87 to 7.47 ng/mL, with IC_50_ being 4.06 ng/mL and 3.74 ng/mL, respectively. The constructed TRFICA method has high specificity towards T&T, and there is no cross-reaction rate with other similar compounds. Importantly, in the diluted milk sample, the LOD of the TRFICA ranged from 1.72 ng/mL to 1.39 ng/mL, with the visual cutoff value being 31.25 ng/mL and 62.50 ng/mL for T&T in milk, respectively. The TRFICA also exhibited high stability at 37 °C. In conclusion, the proposed TRFICA can be regarded as a simple preprocessing method with high specificity, and the detection could be completed in 13 min, which could satisfy the simple, efficient, and specific screening of T&T residues in milk samples. In the future, how to distinguish between T&T independently by improving the TRFICA method will be an interesting research direction.

## Figures and Tables

**Table 1:** Dosage selection of the labeled antibodies for tylosin (*n* = 3).

Labeled Antibodies	1 μL		2 μL		3 μL		4 μL	
Analyst	Negative	125 ng/mL	Negative	125 ng/mL	Negative	125 ng/mL	Negative	125 ng/mL
T/C (M ± SD ^a^)	1.2514 ± 0.12	0.4188 ± 0.23	0.8171 ± 0.14	0.2815 ± 0.08	1.3649 ± 0.18	0.2451 ± 0.07	1.3868 ± 0.19	0.3470 ± 0.04
Inhibition Rate		66.5%		65.5%		82.0%		75.0%

SD ^a^ represents the standard deviation of sample.

**Table 2:** CRs of related or similar compounds in the TRFICA.

Compounds	Chemical Structure	IC_50_ (ng/mL)	Cross-Reactivity (%)
Tylosin	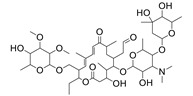	5.76	100
Tilmicosin	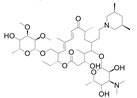	4.63	124.4
Erythromycin	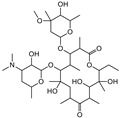	>4000	<0.1
Norfloxacin	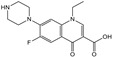	>4000	<0.1
Norfloxacin	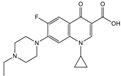	>4000	<0.1
Azithromycin	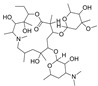	>4000	<0.1
Acetylspramycin	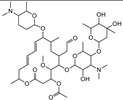	>4000	<0.1
Roxithromycin	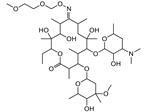	>4000	<0.1
Clinafloxacin	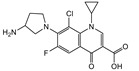	>4000	<0.1
Pefloxacin	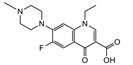	>4000	<0.1
Furazolidone	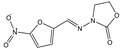	>4000	<0.1
Spiramycin	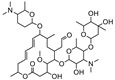	>4000	<0.1
Nitrofural	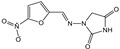	>4000	<0.1
Furadantin	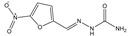	>4000	<0.1

**Table 3:** Stability experiments for TRFICA (*n* = 3) at 37 °C.

Days	Negative Samples	Positive Samples	Inhibition Rate (%)
	(Mean ± SD ^a^)	(Mean ± SD)	
1	1.2523 ± 0.19	0.1188 ± 0.07	90.5
3	1.3621 ± 0.16	0.0872 ± 0.04	93.6
5	1.2398 ± 0.21	0.1002 ± 0.09	91.9
7	1.2478 ± 0.15	0.0983 ± 0.11	92.0

SD ^a^ represents the standard deviation of the measured values.

**Table 4:** Recoveries of tylosin-spiked and tilmicosin-spiked samples (*n* = 3).

			TRFICA			Commercial ELISA Kit		
Samples	Spiked	Spiked Level (ng/mL)	Measured (Mean ± SD) (ng/mL)	Recoveries (%)	CV (%)	Measured (Mean ± SD) (ng/mL)	Recoveries (%)	CV (%)
Milk	Tylosin	5	5.98 ± 0.05	119.6	12.1	6.15 ± 0.08	122.9	1.3
		10	12.45 ± 0.04	124.5	10.1	11.39 ± 0.03	113.9	0.2
		20	24.08 ± 0.18	120.4	1.9	18.41 ± 0.01	92.1	0.1
	Tilmicosin	5	5.56 ± 0.03	111.2	13.9	5.40 ± 0.08	108.1	1.5
		10	8.05 ± 0.02	80.6	7.2	11.21 ± 0.01	112.1	0.1
		20	17.12 ± 0.04	85.7	4.1	19.38 ± 0.01	96.9	0.1

**Table 5:** The results between TRFICA and HPLC-MS/MS for blind samples.

Samples	Analytes	TRFICA (mg/L)	HPLC-MS/MS (mg/L)
Milk 1	Tylosin and Tilmicosin	ND	ND
Milk 2	Tilmicosin	10.99	10.21
Milk 3	Tilmicosin	24.08	25.93

ND: Not detected.

## Data Availability

Data is contained within the article.
